# A Comparative Study on Diadochokinetic Skill of Dyslexic, Stuttering, and Normal Children

**DOI:** 10.1155/2013/165193

**Published:** 2013-08-06

**Authors:** Ayyoub Malek, Shahrokh Amiri, Issa Hekmati, Jaber Pirzadeh, Hossein Gholizadeh

**Affiliations:** ^1^Child and Adolescent Psychiatrist, Clinical Psychiatry Research Center, Tabriz University of Medical Sciences, Tabriz, Iran; ^2^Child and Adolescent Psychiatric Ward, Department of Psychiatry, Razi Mental Hospital, El Goli Boulevard, P.O. Box 5456, Tabriz, Iran; ^3^General Psychology, Shahid Beheshti University, Daneshju Boulevard, P.O. Box 1983963113, Tehran, Iran; ^4^General Psychology, Tarbiat Moallem University, P.O. Box 1571914911, Tehran, Iran; ^5^Clinical Psychology, Tabriz University, P.O. Box 5166614766, Tabriz, Iran

## Abstract

*Objective*. Previous studies have shown some motor deficits among stuttering and dyslexic children. While motor deficits in speech articulation of the stuttering children are among the controversial topics, no study on motor deficits of dyslexic children has been documented to date. *Methods*. 120 children (40 stuttering, 40 dyslexia, and 40 normal) 6–11 years old were matched and compared in terms of diadochokinetic skill. Dyslexia symptoms checklist, reading test, and diadochokinetic task were used as measurement instruments. *Results*. The data analysis showed that there are significant differences (*P* < 0.001) in reaction time and the number of syllables in accomplishing diadochokinetic tasks among stuttering children, dyslexics, and the control group. This indicates that stuttering children and dyslexics have poor performance in reaction time and in the number of monosyllable articulation and long syllable articulation. Furthermore, there are significant differences (*P* < 0.001) in these indices between stuttering children and dyslexics, so that the latter group have better performance than the former one. *Conclusion*. The findings indicate that stuttering children and dyslexics have deficits in diadochokinetic skill which suggests their low performance in the motor control of speech production and articulation. Such deficits might be due to the role of the tongue in the development of stuttering and dyslexia.

## 1. Introduction

Language is the most powerful means of communication. It can manifest as spoken and/or written form [1]. The damage on any aspect of human speech, a part of tongue movement, including (1) the production of verbal sounds, (2) speech speed, and (3) phonemes, can lead to verbal disorder. Likewise, damage to the written and spoken abilities of an individual affects his/her educational and social life [2]. Though language disorder and dyslexia are quite different concepts (e.g., in DSM-IV), some scholars consider them as a continuum of language disorders [3, 4]. Some theories refer to the motor deficit in dyslexia and language disorders. For most children, the multiword language development stage is the onset of stuttering as speech and other motor skills develop. At the age of 2–4, children are able to produce longer and more complex sentences. The rate of speech production increases and their speech rhythm becomes similar to grownups. Simultaneously, their fine and gross motor skills develop. Thus, the ability to acquire new motor skills is vital for this earlier rapid development in language [5]. Children who have a problem in performing complicated motor skills (e.g., speaking) might not be efficient enough in acquiring motor skills. Perhaps, stuttering is because of child's problem in acquiring verbal-motor skill similar to adults [5].

In terms of dyslexia, nowadays this problem is considered beyond pure reading disorder, and the results of most studies show that dyslexic children suffer from such problems as motor skill problem too. The results of the study by Wolf [6] indicate that dyslexic individuals suffer from damages in accomplishing the tasks that require integrity of hand fingers. They argue that poor performance of dyslexic subjects could be due to cognitive inefficiency, the ability to form inner reflection which is necessary for making a movement. Lerner [7] states that 70% of dyslexic children suffer from motor deficits. Among the studies that support his findings are those of Foorman and Torgeston [8] and BabaPour [9]. The results of their studies show motor problems, especially in performing fine movements. 

While most of the aforementioned studies have dealt with fine and gross motor skills, enough attention was not paid to the verbal and speech movement skills such as diadochokinetic skill in stuttering and dyslexic children. Kinesthesia is a modality necessary for movement. Generally, it is defined as the awareness of the position, body, and organ movements accompanied by movement attempts [10]. Diadochokinesia refers to the fast, step by step, changeable, and frequent movement ability of speech production organs as speaker utters different syllables [11]. Verbal diadochokinetic skill is usually measured by maximum repetition rate paradigm and/or the time needed for the oral repetition of monosyllable and multisyllable verbal structures. Indeed, diadochokinetic skill is the time needed to receive and process motor gestures that are necessary for the production of specific and frequent syllables during the time; it is considered as a model for spatial programming of speech [6]. In this type of tasks, meaningless syllables (e.g., one syllable /puh/, /kuh/, and /tuh/ or long syllable /puh-kuh-tuh/ or /pa-ta-ka/) are made by combining vowels and consonants. Studies show that the diadochokinetic skill in children increases as their motor system matures, and at the age of 9-10 or 15, it is similar to adults' motor system, and normal children in performing diadochokinetic tasks make more speech errors in comparison with verbal fluency [12]. 

Few studies have been carried out to investigate stuttering and dyslexic children's diadochokinetic skill. Scott Yaruss and Logan (2002) [12] believe that the findings of studies on diadochokinetic skill among stuttering and dyslexic children are controversial. The majority of these children have a verbal-motor problem which is an index of their performance in accomplishing diadochokinetic tasks. In addition, there are studies that prove stuttering people spend more time to improve the speed of their motor activities. Studies on stuttering people's verbal and motor learning show a low rate of learning meaningless words and slower acquisition of consecutive finger tapping task and syllable reading [13]. Loucks and De Nil (2006) [10], in their study, detected verbal-motor deficit among stuttering adults. The results of their study show that chronic stuttering adults showed less movements and flexibility compared to the control group in the absence of visual feedback. To investigate diadochokinetic skill among the dyslexic children, Fawcett and Nicolson (2002) [14] attempted to find out whether dyslexic children are different from normal ones in speech production, using diadochokinetic tasks. The results of their study show that there is a meaningful difference between dyslexic and normal children only in repeating monosyllables. Moreover, it is proved that the diadochokinetic skill was able to predict reading and reading-related processes directly. 

According to our knowledge, no study has investigated and compared diadochokinetic skill of stuttering and dyslexic children to date. Thus, to fill the gap, the present study aims to detect and compare diadochokinetic ability of stuttering and dyslexic children.

## 2. Methods and Materials

### 2.1. Participants

The study population comprises all the 6–11-year-old stuttering and dyslexic children who referred to child psychiatric or consultation centers in Tabriz. Taking into account the objectives of the study and previous research literature, the total sample size for the present study was calculated as 120 children including 40 stuttering, 40 dyslexic, and 40 normal children. The three groups were matched in terms of age, gender, and bilingualism. The inclusion criteria included being 6–11 years old, being diagnosed as stuttering for stuttering group and dyslexia for dyslexic group, right handedness, the written informed consent signed/sealed by either parents or guardians, being bilingual, and having IQ higher than 90 measured using Raven's IQ test. The exclusion criteria included comorbidity of a psychiatric disorder (children were screened using parent version of Strengths and Difficulties Questionnaire) and suffering from a neurological, sensorimotor, and brain damage. 

### 2.2. Procedure

To accomplish the objectives of the study, having diagnosed as stuttering and dyslexic children by a child psychiatrist and meeting the inclusion criteria, the selected children were tested separately using diadochokinetic task to measure their diadochokinetic skills. In this step, first monosyllables /pa/ta/ and /ka/ were presented one by one orally, and the children were asked to repeat them quickly and in a correct and fluent way. The examiner recorded the time spent for 15 repetitions using a chronometer. To measure the ability to utter the long syllable of /pataka/, the same method was employed and the time spent for 15 repetitions was recorded. In the second step, children were asked to repeat monosyllables and the long syllable separately in 15 seconds. Here, the number of syllables uttered correctly in 15 seconds was recorded. Similar procedures were used for the control group after being interviewed, and it was proved that they meet the inclusion criteria.

### 2.3. Instruments

#### 2.3.1. Dyslexic Symptoms Checklist

It is used to measure the severity of dyslexia. This tool was prepared using the symptoms suggested by the Dyslexia International Association (2003-2004) and the Statistical and Diagnostic Manual of psychiatric disorders, 4th edition (DSM-IV). Cronbach's *α* was used to measure the reliability of the checklist. The estimated reliability was 0.82 which shows that the used checklist was highly reliable. The validity of the checklist was confirmed by the specialists in the “Learning Disability Center” [15]. The checklist was used to measure the severity of dyslexic disorder. 

#### 2.3.2. Pour Etemad Reading Test

To evaluate the dyslexia, the reading test (Pour Etemad 2008) was used. The test had already been administrated to 1500 elementary students from Qum by Hoseinilar et al. [16] to measure its psychometric properties. The test constitutes 11 Farsi texts. Card number 1 is a practice card. Two cards are allocated for each grade. The first text for each grade is a story adopted from a series of stories used in the analysis of “Neal reading ability” test and was rewritten according to the list of vocabularies in Farsi textbook for each grade. The face and content validity of the text were confirmed by the first, second, and third grade teachers. The second text was adopted from the textbook of each grade. To eliminate the memory effect, some words/phrases were replaced with other words/phrases so that the structure of the text changed; however, the meaning remained intact. Time recording was observed in reading texts, but there was no need to record the time for reading the comprehension part. Provided that the child in reading card number 1 did not make more than 24 errors, the second card would be presented. One score was devoted to each mispronunciation (except for analysis and rereading cases) and the spent time was recorded at the end. The scores for reading skill and reading comprehension were measured separately [17].

The statistical analysis of the reading texts refers to their high validity and reliability. Construct validity was estimated by computing correlation coefficient between two sections of the test.  Table 1 shows the construct validity of the cards.

All the computed correlation coefficients were meaningful (*P* < 0.001). In addition, convergent validity in each section of the test was estimated at 0.5 using correlation coefficient between scores, reading Farsi scores and the total scores of reading precision in odd and even cards. It was meaningful at *P* < 0.001 level. Cronbach's *α* and parallel methods were used to measure test reliability.  Table 2 illustrates estimated Cronbach's *α* for reading even and odd cards.

Parallel reliability of odd and even cards for reading precision, reading comprehension, and reading speed was 0.9 [17].

#### 2.3.3. Diadochokinetic Task

It is used to measure diadochokinetic skill of the participants by estimating fine motor skills of children's vocal organs. In Iran, this test is based on the results of Fletcher's test. The test consists of a fixed number of one syllable and long syllable morphemes. It is administrated in two stages. In the first stage, a fixed number of monosyllables /pa/, /ta/, and /ka/ and long syllable /pataka/ were repeated 15 times. In the second stage, the mean of the number of syllables was calculated for fixed 15 seconds in the same order for monosyllables /pa/, /ta/, and /ka/ and three syllables /pataka/. The purpose of the first stage was to measure the number of correct responses, whereas in the second stage, the reaction time was recorded [11]. To administ the test, the examiner repeats syllables to the participant and asks him/her to repeat them as quickly as s/he can. The examiner records the time spent for a specific number of syllables (15 repetitions of monosyllables and 15 repetitions of long syllables). In the second phase, the participant should repeat monosyllables and long syllables in 15 seconds. Fletcher's method was used to record the time needed to mix the sounds. In this method, the examiner uses a chronometer to record the needed time for repeating specific number of syllables. Then, the spent time for uttering all the syllables is recorded as diadochokinetic index. 

#### 2.3.4. Strengths and Difficulties Questionnaire (SDQ)

It is a screening tool used to specify the emotional and behavioral disorders of children and teenagers. It consists of 25 items and evaluates five main psychiatric symptoms of conducting problems, hyperactivity, emotional symptoms, peer relationship problems and prosocial behaviour. Various studies on the psychiatric properties of this tool worldwide report that it is a suitable tool to measure children's and teens' disorders. The questionnaire constitutes three types: teacher, parent, and self-evaluation. In this study, the parent type was used. A study by Tehranidost et al. (2006) showed the appropriateness of this tool in terms of the psychometric properties of the teacher and parent versions in evaluating children's psychiatric disorders. This instrument is used for screening and eliminating children who are afflicted with behaviour disorder at the same time [18].

### 2.4. Data Analysis

SPSS version 17 was used for analyzing the data. MANOVA was used to specify the time gap between the reaction and correct response of diadochokinetic skill among stuttering, dyslexic, and normal children. Pearson's correlation coefficient and multivariable regression were used to investigate the relationship among the severity of dyslexia, reaction time, and the number of correct answers to the diadochokinetic task. 

## 3. Results

Table 3 illustrates the descriptive statistics of diadochokinetic skill among the three groups of participants.

MANOVA was used to measure the difference between dyslexic and normal children's reaction time and the correct response to diadochokinetic task. The results show that Wilks Lambda index at *P* < 0.001, *F* = 69.66 is meaningful, indicating the general effect of the group on diadochokinetic skill. Taking into consideration the significance of variance analysis, the test of between-subject effects was used, the results of which are shown in Table 4.

As Table 4 shows, there is a significance difference (*P* < 0.001) between at least two groups of children in each variable of diadochokinetic skill. Tukey's post hoc test was used to find out the sources of the difference, and the results of which are presented in Table 5.

As illustrated in Table 5, there is a significant difference (*P* < 0.001) between the control group and the dyslexic and stuttering groups in terms of the time they spent to repeat monosyllables and long syllables. Taking into account the mean difference, the control group outperformed the other two groups. Similarly, there is a significant difference between dyslexic and stuttering groups (*P* < 0.001); the dyslexic group outperformed the stuttering group. In other words, the control group in comparison with the two other groups spent less time to utter monosyllables and long syllables. A similar difference was recorded for the stuttering and dyslexic groups; the latter group spent less time compared to the former group. Regarding the number of syllables produced by each group, there is a meaningful difference between the dyslexic and stuttering groups and the normal group. In addition, there is a difference between the stuttering group and the dyslexic group (*P* < 0.001). The mean difference indicates that the control group had significantly articulated the highest number of syllables among three groups of the participants. However, there is a meaningful difference between the dyslexic group and the stuttering group too. In other words, dyslexic children uttered more monosyllables and long syllables compared to the stuttering group. Thus, it could be concluded that both stuttering and dyslexic children's diadochokinetic skills have deficits, which accounts for their poor performance compared to normal children. However, the severity of the deficit in stuttering children was more than in dyslexic children. 

## 4. Discussion

The current study was an attempt to compare diadochokinetic skill of the stuttering and dyslexia children with that of the normal children (control group). The findings indicated that, regarding the time spent on accomplishing diadochokinetic task, the stuttering children spent more time than the other two groups. In terms of the number of monosyllables and long syllables produced by the experimental and control groups, the stuttering children uttered less syllables and made more errors, indicating their poor performance compared to the control group. The results of the present study support the previous findings [19–23]. These studies made use of the different instruments such as nonword repetition sentence [21], diadochokinetic task [19], orofacial ability [20, 23], and the “verbal reaction time” task [22] to show that stuttering children and adults have a problem in verbal-motor control. The high rate of errors in uttering monosyllables and long syllables and spending more time to produce them account for the damaged or undeveloped verbal-motor control of the stuttering people. In a similar vein, according to stuttering psycholinguistic models, the amount of speech articulation is considered as a criterion for verbal-motor skill. It is believed that stuttering people need more time for verbal-motor planning [24]. Cook et al. (2011) believe that though sensory-motor performance deficit may affect other motor systems in stuttering people, verbal-motor system is often damaged selectively [20]. 

Some researchers suggest that stuttering is a sort of motor disorder, and stuttering people have low verbal-motor skill [25]. However, the main deficit might not be attributable to a specific verbal-motor controlling factor. The main problem in detecting the main source of verbal-motor control deficit is that damaged motor process, responsible for the stuttering problem, may be the result of deficiencies in the controlling processes of outside the verbal-motor system programming. Nevertheless, some studies [22] using “verbal reaction time” task came to the conclusion that stuttering people in comparison with normal ones act slowly at the onset of verbal movements. Moreover, phonological and speech analyses of stuttering people provide evidence on lips and jaw movement timing problems [26]. An undisputed assumption is that the problem in tongue processing procedures probably provides verbal movement controlling system with insufficient input, and the stutter, as a result of motor control system, is an attempt to compensate for this weak input [27, 28]. 

Loucks and De Nil (2006) used tendon vibration method with stuttering adults. The results of their study show no general deficit in their perception of the movement condition but a delicate quality difference in unifying this perception. In other words, stuttering people make use of their mouth and jaw position perception less effectively [10]. Alm [29, 30] in his proposed theoretical framework to explain speech production deficit suggests that, at a verbal movement level, the modifications in the automatic neural system reaction can lead to a “frozen response” which manifests at the level of muscle activity and speech production movements. Alm goes further as he has assumed that the error of repeating and lengthening syllables is due to the complicated interaction among motor, emotional, and cognitive factors. 

The results of Saltuklaroglu et al. (2009) showed that the severity of the stutter among stuttering people increased as they were talking and drawing simultaneously in comparison with the situation when they were just drawing or when they were compared to nonstuttering individuals in accomplishing the same task. These levels were on peak when their speech was accompanied by the stutters, and it diminished as the stuttering nearly disappeared. This study confirms the overlap of the stutter and disfluency of hand movements, especially in reading aloud. The findings are in line with a theoretical model which considers the stuttering as a communication disorder that can affect relevant motor performance because of neural connections [31].

In addition, the results of this study indicate that dyslexic children presented poor performance compared to the control group in terms of the number of syllables and the time spent to utter them. Though there are a few studies on the motor skills of dyslexic children's language ability, a study by De Bree et al. [32] investigated the nonword repetition of the children who were at risk of dyslexia. The results showed that stuttering children have problems in repeating nonwords, which supports the finding of the present study. Though nonword repetition task was not exclusively compiled to evaluate the tongue movement skill, since this task deals with speech articulation, the tongue movement factor plays a key role in it. Despite the fact that research on tongue movement is scarce, some studies refer to dyslexic children's deficit in gross and fine movements [8, 9, 33]. Foorman and Torgeston (2001) state that dyslexia is beyond pure reading disorder and dyslexic children show motor deficit, especially in accomplishing fine movements [8]. Some scientists believe that the development and balance between motor skills are the basis of future learning, and what happens during this period affects next learning, especially cognitive acquisition. It seems that dyslexic children have a poor cognitive ability in acquiring motor skills because these skills, especially fine motor skills, require some levels of cognitive activities. It is the reason why some people are slow in acquiring and developing motor activities [9]. One of the theories of motor disorder of dyslexic people is the theory of autonomous-cerebellar dysfunction. According to this theory, autonomous function of motor behaviors is necessary for success in reading fluently, and cerebellum might be important for the development of the autonomous system [14].

Furthermore, the findings of this study indicate that stuttering children's performance in doing the diadochokinetic task was poor compared to that of dyslexic group. Though stuttering children were more inefficient than dyslexic children were, the similarity of motor deficit between the two groups can be explained in the following way. First, the similarities can be due to language mutuality between the two groups. In line with the research literature, some studies [33] argue that learning to read according to the language processes happens at various levels, and language deficits are one of the most common interdependent reading disorder behaviors. Given the fact that some classifications have been suggested for the dyslexic among which are linguistic and perceptual categorization, the present study opted for the former model and focused on children who suffered from linguistic dyslexia. The dyslexic children in comparison with the perceptual group are quick readers who do not pay attention to details and make many mistakes. Most of the studies indicate that children have problems in phonological awareness, speed, and correct reading [34–36], and diadochokinetic skill deficit might be due to language mutuality of discussed disorders. Another explanation for the obtained results could pertain to the fact that we consider the stutters and dyslexics as communicative disorders because as aforementioned, some scholars believe that language is a means of communication, and speech and writing are its outer manifestations [1]. Thus, damage to any aspect of speech and the reading and writing skills, as an aspect of written language, affects individual's interpersonal relationship. It is assumed that speech, considered as vocal tool signs, as ontogenic phenomena developed from hand gestures and communication among our ancestors [37]. It seems that these issues about reading and writing can be discussed as another aspect of language. In addition, the mutual neural basis can account for the motor deficits in both disorders. Finally, the results of this study do not necessarily confirm that dyslexia and stuttering are motor deficits; however, there could be a relationship between motor deficits and these disorders.

## 5. Conclusion

Generally, the results of the present study show that the stutterers and dyslexics present inefficiency in tongue movement, especially in diadochokinetic skill. These problems are more severe among the stuttering group. The deficits indicated that the children had deficits in the motor control of speech control. The similarities between the two groups in terms of motor deficits indicate that the origin of both disorders is the tongue. It could also pertain to the fact that in both disorders similar regions of neural system may have malfunction. In order to be able to detect this dysfunction, the function of the different parts of brain can be scrutinized using fMRI and/or other pieces of equipment as children accomplish diadochokinetic task. It is necessary to mention that, in this study, the stuttering subtypes were not specified. However, it dealt with the linguistic type of dyslexia. These are among the limitations of this study which can be considered in further researches.

## Figures and Tables

**Table 1:** Construct validity of cards.

Construct validity	Even cards	Odd cards
Reading precision	0.6–0.9	0.7–0.9
Reading comprehension	0.3–0.6	0.3–0.5
Reading speed	0.8–0.9	0.8–0.9

**Table 2:** Reliability of cards.

Cronbach's *α*	Even cards	Odd cards
Reading precision	0.9	0.8
Reading comprehension	0.8	0.7
Reading speed	0.9	0.8

**Table 3:** Descriptive indices of diadochokinetic skill among study groups.

Group	Diadochokinetic			
	Monosyllable reaction time	Long syllable reaction time	No. of monosyllables	No. of long syllables
	(sd) M	(sd) M	(sd) M	(sd) M
Dyslexic	(3.88) 25.90	(3.47) 24.95	(11.07) 81.40	12.00 (1.5)
Stuttering	(6.02) 36.15	(3.41) 34.65	(8.14) 69.42	8.77 (1.77)
Normal	(2.80) 20.49	(2.36) 15.94	(9.60) 02.97	14.57 (1.7)

**Table 4:** Between-subject effects of diadochokinetic skill for study groups.

Source of changes	Dependent variable	Sum of squares	df	Mean of square	*F*	Sig.
Group	Reaction time to monosyllables	5058.65	2	2529.32	128.04	0.000
	Reaction time to long syllables	7002.43	2	3501.21	358.33	0.000
	No. of monosyllables	23126.45	2	11536.22	123.32	0.000
	No. of long syllables	675.61	2	337.80	121.70	0.000

Error	Reaction time to monosyllables	2311.22	117	19.75		
	Reaction time to long syllables	1143.17	117	9.77		
	No. of monosyllables	10970.35	117	93.76		
	No. of long syllables	324.75	117	2.77		

**Table 5:** The results of Tukey's posthoc test.

Dependent variable	Group I	Group (J)	Mean difference	St. error	Sig.
Time of monosyllables	Control dyslexic		−5.40	0.99	0.001
	Stuttering		−15.65	0.99	0.001
	Dyslexic stuttering		−10.24	0.99	0.001

Time of long syllables	Control dyslexic		−9.00	0.69	0.001
	stuttering		−18.70	0.69	0.001
	Dyslexic stuttering		−9.70	0.69	0.001

No. of monosyllables	Control dyslexic		21.57	2.17	0.001
	stuttering		33.57	2.17	0.001
	Dyslexic stuttering		11.97	2.17	0.001

No. of long syllables	Control dyslexic		2.57	0.37	0.001
	stuttering		5.80	0.37	0.001
	Dyslexic stuttering		3.22	0.37	0.001
